# Dietary polyunsaturated fatty acids, brain function and mental health

**DOI:** 10.1080/16512235.2017.1281916

**Published:** 2017-02-06

**Authors:** Håvard Bentsen

**Affiliations:** ^a^ Center for Psychopharmacology, Diakonhjemmet Hospital, Oslo, Norway

**Keywords:** lipids, food, psychiatry, depression, psychosis, ADHD

## Abstract

The importance of dietary polyunsaturated fatty acids for our health and our economy is immense. The present paper is a quick guide to this topic, which is highly complex and full of conflicts. One aspect is highlighted, namely the relationship between these substances and our mental health. Relevant chemical and physical properties of polyunsaturated fatty acids are briefly presented. The questions of needs and supplies are discussed. The effects of these fatty acids on the human body, in particular the brain, are described. How do they influence mental health? The most recent meta-analyses and systematic reviews have been used as sources, and outstanding references have been selected. We want to convey updated knowledge, based on good science, for better decision making in preventive and therapeutic health care, as well as in agriculture, fishery and food industries.

## Chemical and physical properties of polyunsaturated fatty acids

Polyunsaturated fatty acids (PUFA) are present in all membranes in our body, ensuring a hydrophobic boundary between hydrophilic compartments.[1,2] They are parts of phospholipids and other complex lipids, esterified to a three-carbon backbone, to which hydrophilic molecules are attached. The hydrophobic parts of these lipids are directed inwards in the bilayer membranes, whereas the hydrophilic parts are exposed to the water phases. In each of the two layers, lipids are non-randomly distributed, e.g. in rafts with specific functions. Proteins and other molecules are embedded in these lipid layers, and the physico-chemical properties of the former depend to a large extent on the quality and quantity of the fatty acids. Fatty acyl chains account for more than half the mass of phospholipids, which account for about 60% of the dry weight of the brain and even more in synapses and dendrites.

The structure of each PUFA is characterised by an acyl chain with one acid end (-COOH) and one methyl end (-CH_3_). PUFA have at least 18 carbon atoms in their chains. Arachidonic acid (ARA) and eicosapentaenoic acid (EPA) have 20, docosahexaenoic acid (DHA) 22. PUFA have at least two double bonds (ARA 4, EPA 5 and DHA 6). «Omega-3» (ω-3, n-3) indicates that the first double bond is found at the third carbon from the methyl end (H_3_C-C-C=), whereas «omega-6» (ω-6, n-6) indicates that the double bond closest to this end is at the sixth carbon. Mammalians need dietary *essential fatty acids*, namely linoleic acid (LA, C18:2) and alpha-linolenic acid (ALA, C18:3), in order to synthesise other PUFA. The ω-6 LA and the ω-3 ALA may be converted into PUFA of the same omega class by unsaturation and elongation (two carbons by each step). PUFA from the two classes compete for the same desaturases and elongases. Thus, the more there is of LA in the diet, the less ALA will be converted into ω-3 long-chain PUFA (i.e. C20-24). The concentration of ARA is less influenced by diet and more tightly regulated, thus less varying in blood and breast milk, than DHA. Decreasing dietary LA will increase EPA, not ARA, whereas increasing dietary ω-3 long-chain PUFA may decrease ARA.[3]

The most abundant PUFA in the brain are ARA (ω-6) and DHA (ω-3), accounting for about one fifth of the brain’s dry weight. The amount of another important ω-3 PUFA, EPA, is small because of extensive metabolism. Both ARA and EPA may be transformed into various eicosanoids (i.e. molecules containing 20 carbon atoms) of great functional importance. DHA may be transformed into docosanoids. The double bonds (-C = C-) must have a *cis*-configuration (-_C_/^C=C^\_C-_) to be physiologically active. This entails a rounded shape of the molecules. The polyunsaturated structure (=CH-CH_2_-CH=) makes rotation about the single bonds (C-C) adjacent to double bonds much more likely than if there were no such double bonds. This increases the flexibility of the molecule tremendously. Both the round shape and the flexibility lower the melting point and makes membranes more fluid.

The methyl groups between the carbons with   double bonds (=CH-CH_2_-CH=) are prone to peroxidation, by which a reactive oxygen or nitrogen substance or a free radical abstracts one hydrogen atom, leaving and remove one unpaired electron on the carbon atom. This carbon-centred radical may react with O_2_ to make peroxyl radicals, propagating a chain reaction of lipid peroxidation. In the absence of O_2_, carbon radicals might cross-link. These reactions can be interrupted by antioxidants. It is crucial for the protection of PUFA that sufficient antioxidants are present, derived from synthesis in the body and from the diet.

## In what foods are PUFA found?

Industrial production of food has caused a marked increase in the ω-6/ω-3 ratio. The original ratio in the human diet was probably 1:1–2:1, whereas a typical current Western diet has a ratio of 10–20:1.[4] Both ARA and DHA are needed for the development and the function of the brain, but the current imbalance may indicate that the amount of dietary ω-6 PUFA should be reduced and the amount of ω-3 should be increased. Daily recommended intake of EPA+DHA is at least 250 mg. Typically, LA is found in seeds and oil from corn, sunflower, soya and wheat. ARA is found in animal sources, such as eggs and meat, depending on the amount of LA and ALA in the fodder. Examples of rich sources of ALA are flax, chia, lingonberry (all about 50% ALA of total oil), walnut and rapeseed (both 10%). The richest sources of ω-3 long-chain PUFA (EPA, docosapentaenoic acid and DHA) are oily fish, fish oil and cod-liver oil. However, wild fish stocks have declined dramatically in the last 70 years, and they approach extinction within 20–30 years if business-as-usual management of fisheries continues.[5] They may be rescued within 10 years under alternative policies. Aquaculture of fish and shell-fish has increased steadily the last 35 years, mostly in Asia, and is now yielding more fish than fisheries.[6] Culture and fermentation of microalgae may produce specific ω-3 fatty acids. Meat, milk and eggs from domestic animals may be enriched in ω-3 PUFA by changing the fodder (e.g. cattle fed grass and legumes, chicken fed linseed and rapeseed oil).[7] According to recent meta-analyses, organic milk and meat contain 50% more ω-3 PUFA than conventional alternatives.[8,9] The value of plants as a source of ω-3 long-chain PUFA has been underestimated because of deficient scientific methods.[10] Vegans may have sufficient concentrations of EPA and DHA in their body to function at a high mental level.

## The effects of PUFA on the human body

The physiologic functions of PUFA fatty acids may be summarised as follows.[2,11,12] (1) *Enhance oxidation*. PUFA in triacylglycerol may serve as fuel. They regulate transcription factors, thereby enhancing fatty acid oxidation. (2) *Constitute and modulate membranes*. They increase fluidity. They optimise position, amount and function of membrane proteins. (3) *Enhance signal transmission*. This requires that PUFA have been liberated from the membrane by phospholipases. PUFA regulate gene expression directly or through substances in the cytosol. PUFA serve as precursors to second messengers (eicosanoids, docosanoids). Thereby, they regulate inflammation, immunity, blood vessels, platelets, synaptic plasticity, cellular growth, pain, sleep, etc. In general, ω-3 fatty acids *inhibit* whereas ω-6 fatty acids *enhance* inflammation. Conventionally up to 3 g per day of EPA+DHA have been considered safe. Exaggerated intake of ω-3 long-chain fatty acids will have detrimental health effects, and older or diseased persons tolerate less.[13] Effects may include loss of microbial immunity, increased susceptibility to chronic infections and inflammatory bowel disease, colon and prostate cancer, atrial fibrillation and nervous system disturbance. A common mechanism may be that excessive ω-3 long-chain PUFA, especially DHA, disrupt lipid rafts in plasma membranes, promoting displacement of proteins, thereby altering signal transmission.

The brain is particularly rich in long-chain PUFA, especially in excitable membranes. DHA constitutes about 20% of the brain’s fatty acids, whereas ARA accounts for about 15%. The accumulation of these fatty acids in the brain is most intense during the third trimester of pregnancy and the first two years after birth, when it is plateauing.[14,15] At birth, the brain is structurally fully developed, but has reached only 25% of its adult volume. After birth, glial cells and neurons’ axons and dendrites expand and nerve fibres are myelinated. This growth depends heavily upon the presence of long-chain PUFA. Thus, dietary deficiencies of these fatty acids during the brain growth spurt impair neurodevelopment and may cause permanent mental deficits. The development and function of the important monoaminergic systems, such as serotonergic, dopaminergic and noradrenergic pathways, are closely associated to long-chain PUFA.[16–18] Synaptic plasticity is enhanced by PUFA,[19] and both ARA and DHA stimulate neurogenesis in the hippocampus.[20,21]

## The effects of PUFA on mental health

### Normal functioning

There is no conclusive evidence that maternal supplementation with ω-3 long-chain PUFA during pregnancy improves cognitive or visual development.[22] However, one good study showed that such treatment may hinder developmental delay (IQ<85).[23] There is strong evidence, also from a large randomised controlled trial, that breastfeeding improves cognitive development.[24] PUFA are essential constituents of breast milk, and it is likely that they contribute to this beneficial effect. Nevertheless, there is still no good evidence that supplementation of long-chain PUFA to breastfeeding mothers enhances neurodevelopment.[25] Probably, this effect depends on the mother’s PUFA status at baseline.

A recent meta-analysis of the cognitive effects of ω-3 PUFA supplementation in the general population indicated no significant benefit.[26] The authors did not analyse in an appropriate way whether the effects depend on the dose of EPA, which is likely. One trial, performed in 59 moderately malnourished Mexican school-aged children, stands out with remarkable results.[27] A three-month randomised double-blind intervention with EPA 270 mg + DHA 180 mg or placebo (soybean oil) showed that ω-3 PUFA yielded significantly better effect in measures of IQ, executive functioning, visuoperceptive capacity and processing speed. Across 19 tests, changes of large effect size were obtained by a median of 73% among ω-3 children versus 25% of the placebo children. This trial differed from the others in the Cooper et al. [26] paper by being performed in a group with a very low intake of sea food. The baseline and the dose matter.

A dose-dependent effect of fish intake on neurocognitive functioning have been shown in a cross-sectional Norwegian study in healthy elderly people (*n* = 2031).[28] Ten g or more per day of fish protected against poor performance. The optimal performance was reached at 75 g fish per day, corresponding to two fish meals per week. Beyond this amount, the performance plateaued. Lean fish was at least as effective as fatty fish, which could imply that other substances than ω-3 PUFA were decisive.

However, a well-performed 26-week randomised controlled trial with moderately large doses of ω-3 PUFA (EPA 1.3 g day^–1^ + DHA 0.9 g day^–1^) in 65 healthy older adults indicates that these fatty acids have indeed beneficial effects on the brain and cognition.[29] Executive function improved significantly. This was linked to increases in omega-3 index and brain derived neurotrophic factor. In addition, the treatment prevented a decrease in grey matter volume observed in the placebo group, and it had beneficial effects on white matter microstructural integrity, carotid intima media thickness and diastolic blood pressure. The authors conclude, ’Our findings suggest novel strategies to maintain cognitive functions into old age’ (p. 3059).

### Prevention or treatment of disease

#### Affective disorders

In a recent meta-analysis, data from 31 observational studies, over 255,000 individuals and over 20,000 subjects with depression were examined.[30] It shows a 20% lower risk of depression among those with highest quintile compared to lowest quintile of fish or ω-3 PUFA intake. A dose-response analysis revealed a J-shaped association with a peak decreased risk for 1.8 g day^–1^ intake of ω-3 PUFA (relative risk = 0.30, 95% confidence interval = 0.09–0.98). Dietary ω-3 PUFA should be seen in relation to dietary ω-6 PUFA. Probably increasing the former and lowering the latter will yield an optimal protection against depressive disorders, comparably to the effect on chronic headache.[3,31]

People with depressive disorder have lower levels of ω-3 PUFA than healthy controls.[32] In the most recent meta-analysis of long-chain PUFA for depression, 35 randomised controlled trials including 6665 participants receiving ω-3 long-chain PUFA and 4373 participants receiving placebo were examined.[33] Among participants with diagnosed depression, EPA-predominant formulations (>50% EPA) demonstrated clinical benefits compared with placebo (Hedge’s G = 0.61, *p* < 0.001). This is a moderately large effect size, greater than that seen for pharmaceutical antidepressant trials (weighted effect size = 0.37, CI% 0.33–0.41, published studies).[34] DHA-predominant formulations (>50% DHA) did not perform better than placebo. EPA failed to prevent depressive symptoms among populations not diagnosed for depression. In a meta-regression study, Mocking et al. [35] have shown that the effect on depression depends on the dose of EPA, not on the ratio EPA/DHA.

#### Psychoses, non-affective

In a remarkable randomised controlled trial among 81 ultra-high risk adolescents, Amminger et al. [36] have shown that EPA 0.70 + DHA 0.48 g day^–1^ + α-tocopherol 30 mg day^–1^ for 12 weeks prevented transition into psychosis. Four out of 41 in the ω-3 group against 16 out of 40 in the placebo group developed a psychotic disorder. The risk difference was 30% (CI 95% 10–50), *p* = 0.002. This difference was maintained during an eight-year follow-up period.[36] The authors discuss two plausible mechanisms: antioxidants given during a critical period may have an enduring neuroprotective effect (Comment: the combination of ω-3 long-chain PUFA and α-tocopherol in therapeutic doses may have been decisive); or a reduction of dopamine synthesis (Comment: a less convincing explanation).

In established schizophrenia, levels of both DHA and ARA are lower than in healthy controls, and this difference is greater for those who have never used antipsychotic drugs.[37] Fatty acids, including EPA, are inefficient against symptoms in schizophrenia.[38] In a randomised controlled trial, Bentsen et al. [39] showed that EPA 2 g day^–1^ may have a highly significant psychotogenic effect in patients with low levels of red blood cell PUFA during an acute phase. If given together with vitamin E 364 mg day^–1^ + vitamin C 1000 mg day^–1^, this detrimental effect is avoided (*p* = 0.007). We interpret the low PUFA levels at baseline as a protective reaction to oxidative stress, which is probably due to an inherent redox regulatory deficiency. Giving EPA to these patients will further increase the oxidative stress, provoking a worsening of psychosis. Combining the PUFA and the antioxidant vitamins effectively hinders the worsening. This resembles nature, where PUFA are always associated with sufficient amount of antioxidants to protect them from peroxidation.

#### Hyperkinetic disorders (ADHD)

ADHD is associated with a Western dietary pattern, characterised by more saturated fat, sugar and sodium, and less ω-3 fatty acids, fibre and folate, in contrast to a healthy dietary pattern.[40] ADHD is twice as likely if the subject adheres to an unhealthy diet. However, there is no negative link with a healthy diet. Thus, according to these data, removing toxic substances should be given priority to adding protective or attenuating substances.

Levels of ω-3 fatty acids, especially DHA, are lower in ADHD.[41] This baseline level should be taken into account when treating. Omega-3 supplementation is more effective in those with low levels at baseline. A meta-analysis of 16 randomised trials shows that parents and teachers report improvement of hyperactivity/impulsivity in young ADHD patients treated with ω-3 fatty acids (g = 0.26, CI95% 0.15–0.37).[41] Parents also report improved attention. The higher the dose of EPA, the better is the effect on ADHD.

#### Cognitive deficits

Blood levels of ω-3 long-chain PUFA are lower in dementia, whereas only EPA is lower in patients with minor cognitive deficits.[42] Observational studies show that fish and ω-3 PUFA are linked to less risk of cognitive decline and dementia.[43] A meta-analysis of randomised controlled trials with and ω-3 PUFA suggests an effect of n-3 FAs within specific cognitive domains in patients with minor cognitive deficits, but not in Alzheimer’s disease subjects. Briefly, Alzheimer’s disease should be prevented, because it cannot be treated efficiently. Omega-3 fatty acids, a Mediterranean-like diet and exercise may be elements in such a lifestyle intervention, which should start decades before dementia is expected to appear.

## Conclusion

Long-chain PUFA are potent, but vulnerable, general cellular modulators. They are especially important for the development, maintenance, and function of the nervous system. They make membranes more fluid and act as signal substances, directly or via metabolites. Omega-3 fatty acids are mainly anti-inflammatory, omega-6 fatty acids are mainly pro-inflammatory. Omega-3 long-chain fatty acids can prevent and treat mental disorders. The evidence is best for affective disorders. The amount and effects of fatty acids depend on other nutrients and lifestyle factors. Think holistically! We eat too much omega-6 fatty acids compared to omega-3 fatty acids. However, marine sources of omega-3 fatty acids are endangered. Developing sustainable sources of these nutrients is a lifesaving challenge for science and industry.
